# ST-Elevation Myocardial Infarction After Sumitriptan Ingestion in Patient with Normal Coronary Arteries

**DOI:** 10.5811/westjem.2015.6.25920

**Published:** 2015-10-20

**Authors:** Christian Jensen, Mark Riddle

**Affiliations:** Carl R. Darnall Army Medical Center, Department of Emergency Medicine, Fort Hood, Texas

## Abstract

Sumitriptan has been used by millions as a migraine abortant; however, there have been studies showing angina pectoris, coronary vasospasm, and even myocardial infarction in patients with predisposing cardiac risk factors. The majority are patients using the injectable form subcutaneously. We present the case of a patient who presents with ST-elevation myocardial infarction, with no cardiovascular risk factors, after ingesting oral sumitriptan for her typical migraine.

## INTRODUCTION

Injectable triptans used for abortive migraine therapy have been on the U.S. market since 1993. They have been associated with instances of chest pain, coronary vasospasm, and myocardial infarction; but rarely have serious adverse events with oral triptans been reported in literature.

Patients with acute coronary syndrome, which includes ST-elevation myocardial infarction (STEMI), Non-STEMI, and unstable angina, present to emergency departments (EDs) in the U.S. and abroad frequently. In the last decade EDs have made great advances in decreased mortality and morbidity for these patients. Those advances include decreased time to coronary catheterization, use of thrombolytics, and access to emergency medical services (EMS).

We present the case of a patient who developed STEMI one hour after ingesting sumitriptan for her typical migraine. Nitroglycerine was administered by EMS, which helped relieve the coronary artery vasospasm that was causing the myocardial infarction. Triptan-induced vasospasm and infarction must be considered in patients with recent migraine treatment, even in those without cardiac risk factors.

## CASE REPORT

A 49-year-old Caucasian female presented to a community ED by EMS after having abrupt onset chest pain following ingestion of sumitriptan for migraine. She reportedly took sumitriptan orally approximately 60 minutes prior to treat the typical symptoms of her migraine, which she has had intermittently for years. She had taken sumitriptan multiple times in the past without incident. Shortly after taking her medication she had an acute onset of sub-sternal chest pressure, which radiated to her jaw. This pain started at rest and had never occurred before.

She had a past medical history of migraine and depression, for which she took sumitriptan and desvenlafaxine, respectively. Desvenlafaxine is a serotonin–norepinephrine reuptake inhibitor (SNRI) that she has been taking for years. Her last dose of sumitriptan prior to the incident was several weeks before. She had no history of coronary artery disease (CAD), diabetes mellitus, pulmonary disorders, tobacco abuse, cocaine use, or any recent illness or injury. She did not take exogenous estrogen nor had any family history of heart disease.

**Figure 1:**
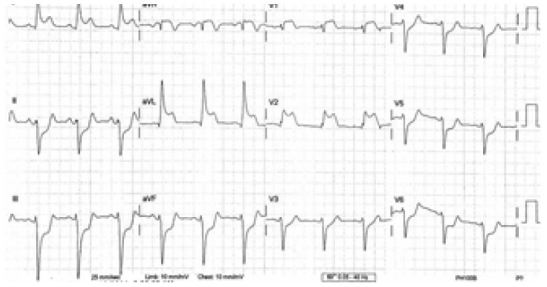
Initial emergency medical services electrocardiogram showing ST-segment elevations across precordial leads consistent with anterior ST-elevation myocardial infarction with reciprocal changes.

She called EMS after having 30 minutes of constant chest pain that radiated to her jaw. She was assessed by the local EMS crew and was given 324mg aspirin PO and 0.4mg nitroglycerine sublingually. Her initial EMS 12-lead electrocardiogram (ECG) showed ST elevations in I, aVL, V1, and V2. She also had ST depressions in II, III, aVF, and V3–V6 (Figure 1). The ECG was transmitted electronically to the ED. The emergency physician interpreted the ECG as a likely anterior myocardial infarction with reciprocal changes in the inferior and lateral leads. The cardiac catheterization lab was activated and the cardiologist on call contacted.

**Figure 2:**
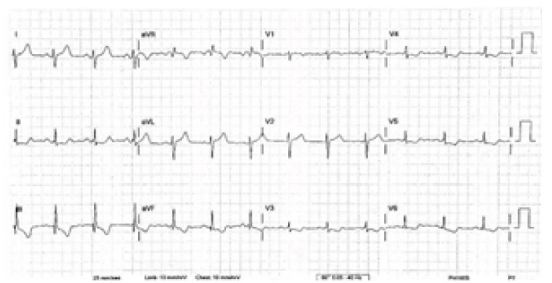
Post-nitroglycerine electrocardiogram with interval improvement of ST-elevation myocardial infarction.

**Figure 3:**
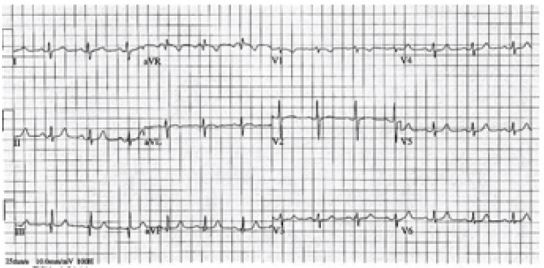
Post-nitroglycerine electrocardiogram with resolution of ST-elevation myocardial infarction.

During patient transport, her pain gradually improved after administration of the nitroglycerine and a second ECG was electronically transmitted (Figure 2) which showed some improvement in the ischemic changes. Once she arrived to the ED, her chest pain had nearly resolved, she had stable vitals and her arrival ED ECG showed resolution of ischemic changes (Figure 3). Cardiac enzymes showed an initial troponin of 0.05ng/mL. Urine drug screen was negative, confirming that no recreational drug use, to include cocaine, was used. Cardiology was present in the ED and elected to take the patient for emergent coronary angiography.

Coronary angiography demonstrated severe constriction of the left anterior descending artery responsive to intracoronary nitroglycerin. There were no lesions suggesting CAD. The left ventricular systolic function was normal with an ejection fraction of 60%. She was diagnosed with severe spasms of the left anterior descending artery leading to myocardial infarction. The patient was transferred to a step-down bed and discharged from the hospital the next morning. The patient’s cardiologist advised her to avoid all anti-migraine medication and to use sublingual nitroglycerin tablets as directed to prevent further angina.

## DISCUSSION

Sumitriptan belongs to the anti-migraine medication class called the triptans, which targets the 5-hydroxytryptamine (5-HT1) serotonin receptor in vascular smooth muscle. Initially, these medications were believed to abort migraines by targeting the vasoconstricting 5-HT1 receptors solely in the cerebral vasculature.^1^ Coronary circulation was believed to possess only serotonin 5-HT2 receptors, ensuring that coronary vasoconstriction would be avoided in triptan use. Despite this, there have been studies showing vasoconstrictive effects in the coronary circulation with the injectable form of these medications.^2^ There have been few reports of patients having myocardial ischemia or infarction with the oral form of sumatriptan,^5^,^9^ as in this case, with even fewer showing coronary angiographic evidence of coronary spasm.^6^

Based on the limited evidence, it is recommended that triptans be avoided in patients with a history of Prinzmetal’s angina, uncontrolled hypertension, and ischemic stroke or heart disease.^7^ Variant angina (VA), which is also referred to as Prinzmetal angina, is a condition characterized by episodes of angina pectoris, usually at rest and can have an association with ST-segment elevation on the ECG, both of which were present on our patient.^11^ The coronary artery vasospasm, caused by spasm of the smooth muscle layer of the arterial wall, generally occurs in the absence of high grade coronary artery stenosis.^10^ The transient myocardial ischemia will cause angina and myocardial infarction can occur in some cases. Spasm can occur in the absence of any preceding increase in myocardial oxygen demand, as was the case with our patient whose pain started at rest. Spasm can occur in angiographically normal coronary vessels, as in our patient, or at the site of atherosclerotic plaques of varying severity.^4^

Multiple drugs including ephedrine-based products, cocaine, marijuana, alcohol, butane, and amphetamines often accompany episodes.^3^,^4^ VA can also be associated with other vasospastic disorders, such as Raynaud’s phenomenon.^8^ Myocardial infarction can lead to life-threatening arrhythmia and is usually due to concurrent obstructive CAD. The fact that our patient had no lesions on coronary angiography is uncommon.

A recent study by Acikel et al. in 2010,^12^ described a similar case of a 48-year-old woman who presented with Prinzmetal-VA with diffuse ST-segment elevation on the ECG. That patient was using zolmitriptan and citalopram. They proposed a correlation between the vasospasm caused by a triptan and the possible increased risk of vasospasm caused by elevated serotonin levels in the plasma from the selective serotonin reuptake inhibitors (SSRI).^10^ The patient in their case had a cardiac risk factor of chronic tobacco abuse, which was absent in our case. The zolmitriptan was taken 10 hours prior to the onset of her chest pain, where the sumitriptan in our case was taken within the hour. Their patient’s ECG showed diffuse ST-elevations, where ours was localized to the anterior leads with reciprocal changes in the inferior leads.

## CONCLUSION

In summary, patients should be counseled on the potential cardiovascular risks of sumitriptan, even if there is no prior history of CAD. If there are cardiac risk factors, this medication should be avoided, or first attempted under close medical supervision. This case should also make providers aware of the possible additive effects of triptans and SSRI/SNRIs when it comes to cardiovascular disease. Noninvasive cardiac imagining like computed tomography or magnetic resonance angiography may play a future role in screening patients to determine if it is potentially safe to use sumitriptan or other anti-migraine medications. This case enforces the need for timely EMS response and early transmission of ECGs to emergency physicians.
